# Guvermectin, a novel plant growth regulator, can promote the growth and high temperature tolerance of maize

**DOI:** 10.3389/fpls.2022.1025634

**Published:** 2022-10-14

**Authors:** Borui Zhang, Huige Gao, Guozhen Wang, Sicong Zhang, Mengru Shi, Yun Li, Zhongqiao Huang, Wensheng Xiang, Wenna Gao, Can Zhang, Xili Liu

**Affiliations:** ^1^ Department of Plant Pathology, China Agricultural University, Beijing, China; ^2^ State Key Laboratory for Biology of Plant Diseases and Insect Pests, Institute of Plant Protection, Chinese Academy of Agricultural Sciences, Beijing, China; ^3^ Science and Technology Research Center of China Customs, Beijing, China; ^4^ College of Plant Protection, Northwest A&F University, Yangling, China

**Keywords:** maize, guvermectin, heat temperature response, plant growth regulator, small heat shock protein

## Abstract

Guvermectin is a recently discovered microbial N9-glucoside cytokinin compound extracted from *Streptomyces sanjiangensis* NEAU6. Although some research has reported that N9-glucoside cytokinin compounds do not have the activity of cytokinin, it has been noted that guvermectin can promote growth and antifungal activity in *Arabidopsis*. Maize is an important food crop in the world and exploring the effect of guvermectin on this crop could help its cultivation in regions with adverse environmental conditions such as a high temperature. Here, we investigated the effects of guvermectin seed soaking treatment on the growth of maize at the seedlings stage and its yield attributes with different temperature stresses. The maize (cv. Zhengdan 958) with guvermectin seed soaking treatment were in two systems: paper roll culture and field conditions. Guvermectin seed soaking treated plants had increased plant height, root length, and mesocotyl length at the seedlings stage, and spike weight at maturity in the field. But only root length was increased at the paper roll culture by guvermectin seed soaking treatment. Guvermectin seed soaking treatment reduced the adverse effects on maize seedling when grow at a high temperature. Further experiments showed that, in high temperature conditions, guvermectin treatment promoted the accumulation of heat shock protein (HSP) 17.0, HSP 17.4 and HSP 17.9 in maize roots. Comparative transcriptomic profiling showed there were 33 common differentially expressed genes (DEGs) in guvermectin treated plants under high temperature and room temperature conditions. The DEGs suggested that guvermectin treatment led to the differential modulation of several transcripts mainly related with plant defense, stress response, and terpenoid biosynthesis. Taken together, these results suggested that the guvermectin treatment promoted the growth and tolerance of high temperature stresses, possibly by activation of related pathways. These results show that guvermectin is a novel plant growth regulator and could be developed as an application to maize seeds to promote growth in high temperature environments.

## Introduction

Temperature plays an important role in the growth and flowering of plants. When the temperature is in an optimal range, plant growth and flowering are accelerated (Granier et al., 2002; Balasubramanian et al., 2006; Nagel et al., 2009). When a critical temperature threshold is exceeded, plant growth can be retarded, and continued exposure can lead to cell death (Hatfield et al., 2011; Gray and Brady, 2016). Furthermore, the high temperature stress causes pollen vigor and a decrease in seed number (Kim et al., 1996; Whittle et al., 2009; Siebers et al., 2015). The effect of high temperature on photosynthesis can also be dramatic. Rubisco activity is decreased, and even inactivated under high temperatures (Crafts-Brandner, 2002; Allakhverdiev et al., 2008). On the cellular level, high temperature can affect the stability of proteins and membranes (Scharf et al., 2012; Hu et al., 2015), induce the accumulation of reactive oxygen species (ROS) (Petrov et al., 2015; Yao et al., 2017; Muhlemann et al., 2018), alter plant hormone production and signaling (Castroverde and Dina, 2021; Li et al., 2021), and induce transcriptomic and metabolomic changes. For tolerating this, when a plant senses these changes on a cellular level under high temperature, it can initiate cellular and metabolic responses that enable it to adapt to these conditions. This includes initiation of signal transduction networks that regulate the expression of a series of genes, such as those encoding heat shock proteins (HSPs) (Pareek et al., 1995; Shah et al., 2011; Bita and Gerats, 2013), accumulating signal, such as nitric oxide, hydrogen sulfide, ROS and stabilizing the cell membranes (Xuan et al., 2010; Li et al., 2014; Li et al., 2018; Qi et al., 2019), therefore increasing the high temperature tolerance of the plant.

Plant growth regulators (PGRs) are agents for the regulation of plant growth and the response to biotic/abiotic stress (Zulfiqar et al., 2019; Zulfiqar and Ashraf, 2021; Zulfiqar and Ashraf, 2022). To accelerate and maintain the growth and production of crops under different environmental factors, PGRs are heavily used in agribusiness (Rademacher, 2015).

Application of PGRs uniconazole, 6-benzylaminopurine, and brassinolide can inhibit senescence in maize leaves, increasing the rate of photosynthesis and crop production (Gao et al., 2017; Ahmad et al., 2019). Gibberellic acid 3 (GA-3) could improve seed germination, seedling growth and the tolerance of water stress as a common maize seed treatment agent (El-antably, 1974; Al-Shaheen and Soh, 2016). Abscisic acid (ABA) and triadimefon could improve the high temperature tolerance of roots and the aerial parts of maize by increasing tolerance to heat stress (Bonham-Smith et al., 1988; Gong et al., 1998). In addition, exogenously applied ascorbic acid, alpha-tocopherol, brassinosteroids, methyl jasmonates, and triazoles can enhance rice production and tolerance to high temperatures (Fahad et al., 2016). Among them, triazoles could impact on the tolerance of stress by the balance of some plant hormones, such as cytokinin, ABA, ethylene and GA (Rademacher et al., 1987; Grossmann et al., 1989; Grossmann et al., 1994; Zhang et al., 2020). However, inappropriate use of PGRs can lead to inhibition of crop plant growth. Application of 1-aminocyclopropane-1-carboxylic acid or 2-aminoethoxyvinyl glycine, inhibits root elongation (Alarcón et al., 2009) and treatment with triazoles can affect seed emergence and seedling growth under low temperatures (Zhang et al., 2020).

Recently, guvermectin has completed registration as plant growth regulators in China. Guvermectin, which is an N9-glucoside cytokinin conjugate, was extracted from *Streptomyces sanjiangensis* NEAU6 (Xiang et al., 2020). Research on N9-glucoside cytokinin conjugates was historically halted due to early conclusions that they were inactive in plants (Vylíčilová et al., 2020). Although N9-glucoside cytokinin conjugates are structurally similar to other cytokinins, the cytokinin activity of naturally occurring N9-glucoside cytokinin conjugates is decreased or completely inactive. No N9-glucoside cytokinin compounds significantly activated either of the *A. thaliana* CRE1/AHK4 or AHK3 CK receptors (Doležal et al., 2007). Interestingly, however, it was reported that these compounds possessed anti-senescent activity in plant bioassays (Matušková et al., 2020). The results implied that N9-glucoside cytokinin compounds were a kind of plant growth regulator with a novel mechanism different to that of cytokinins. As such, guvermectin, an N9-substituted allulose of aminopurine, accelerated the growth of *Arabidopsis* when applied by seed soaking or spraying (Yang and Xiang, 2021; Zhang and Xiang, 2021). Until now, little was known about the effect of guvermectin treatment on crop growth and the mechanism through which it acts.

Maize is one of the most important food crops in the world (Shiferaw et al., 2011).The objectives of this study were to determine the effects of guvermectin seed soaking treatment on maize growth at the seedling stage, yield attributes, and maize seedling growth under high temperature stress. The expression of small HSPs was used as an indicator for the tolerance of high temperature. Furthermore, transcriptomic analysis was used to explore the mechanism of guvermectin. The findings of the present study will strengthen our understanding for the application of guvermectin to crops and aid in the development of efficient plant growth regulators.

## Materials and methods

### Chemicals and plant materials

Chemicals included 70% guvermectin given from Prof. Xiang lab of Northeast Agricultural University, Gibberellic acid 3 (GA3) (90%, BR, Heowns, Tianjin, China), and Antiformin (Sodium hypochlorite aqueous solution, Hushi, Shanghai). These chemicals were stored at 4°C in the dark. Seeds of maize cv. Zhengdan 958 were purchased from China Shandong Shouguang jingguo Co., Ltd. Seeds of maize cv. B73 were given from the National Maize Improvement Center of China Agriculture University.

### Growth conditions in paper roll culture system and field

In paper roll culture, the seeds (cv. Zhengdan 958) were sterilized in 3% NaClO for 10 min, followed by washing with sterilized water three times. Then the seeds were imbibed in 50 mg/L guvermectin in the dark for 24 h. Control seeds were imbibed in 0 mg/L guvermectin at 25°C in the dark for 24 h. The soaked seeds were sowed on 3 layers of wet rolled seed germination paper in a bag and germinated at 25°C in the dark. After 4 days, 7 days, and 14 days, root length and shoot length were measured. The concentrations of 50 mg/L guvermectin in paper roll culture was determined according to our field test.

In the field, the seeds were sterilized in 3% NaClO for 10 min, followed by washing with sterilized water three times. Then the seeds were imbibed in 0 mg/L, 30 mg/L, 50 mg/L, 70 mg/L guvermectin or 50 mg/L GA-3 in the dark for 24 h. Previous study reported that 50 mg/L GA-3 can improve the seed germination, seedling growth (El-antably, 1974). So, 50 mg/L GA-3 seed soaking treatment was used as PGR control. The maize seeds (cv. Zhengdan 958) soaked in different concentrations of guvermectin or GA-3 were sown in the summer of 2018 in the experimental field of the Shangzhuang, China Agriculture University, China (40° 8’N 116° 11’E). A total of 120 seeds were sown in 60 planting pits per block, with 4 random blocks per treatment, total 20 blocks. After 4 days and 7 days, the germination potential were measured.)After 7 days, the plant height, root length, and mesocotyl length were measured. After 14 days, the plant height, root length, and mesocotyl length and stem diameter were measured. After 85 days, at maturity, the plant height, stem diameter, ear height, spike weight per block, spike number per block, and the 1000-grain weight were measured.

### Varying temperature treatments in paper roll culture system

The seeds were treated according to above mentioned methods in paper roll culture. For comparing the influence of different temperature stress, three temperature treatments were set. For chill stress, 1-day-old seedlings (cv. Zhengdan 958) by 50 mg/L guvermectin or 0 mg/L guvermectin treatment were transferred to 4°C for 3 days, and then transferred into the 25 °C growth chamber in the dark until 7 days. For cold stress, 1-day-old seedlings (cv. Zhengdan 958) by 50 mg/L guvermectin or 0 mg/L guvermectin treatment were transferred to 10°C for 3 days, and then transferred into the 25°C growth chamber in the dark until 7 days. For heat temperature stress, 4-day-old seedlings (cv. Zhengdan 958) by 50 mg/L guvermectin or 0 mg/L guvermectin treatment were transferred to 47°C for 15 hours, and then transferred into the 25°C growth chamber in dark for 2 days until 7 days. After 7 days, root length and shoot length were measured.

### Measurement of leaves surface temperature in pot culture under high temperature stress

Due to the seedlings difficultly grow to the three-leaf stage in paper roll culture, we change the pot culture as culture system. The seeds were treated according to the above methods in paper roll culture. The soaked seeds (cv. Zhengdan 958) were sowed in plastic planters containing 1:1 vermiculite and peat moss. After seed germination for 14 days, the three-leaf stage plants of guvermectin treatment were transferred into 47°C climate chamber in the dark for 15 h. The surface temperature of leaves was measured by VarioCAM HD infrared camera (InfraTec).

### RNA extraction and quantitative PCR

Collocated the 4-day-old seedlings (pre-heat treatment), 5-day-old seedlings (after heat treatment for 15 h) and 7-day-old seedlings (recovered 2 days at room temperature). The roots and shoots of seedlings (cv. Zhengdan 958) cultured under the described high temperature conditions were ground in the mortar and frozen in liquid nitrogen. Total RNA was isolated with the Promega RNA extraction kit. Complementary DNA was then synthesized with the ABM RNA one-step reverse transcript kit. Quantitative PCR (qPCR) was carried out using the SYBR green PCR master mix. The quantification of transcripts was normalized to 18s RNA.

The qPCR primer sequences used were:

*HSP 17* 5’-AAGAAGCCCAAGACCATCGA-3’ and 5’-CCCAGACGACACATCAGGTA-3’
*HSP 17.4* 5’-CTGCAAGTTCTGGCCATGAG-3’ and 5’-CGGTCGGATACAGTCAGTCT-3’
*HSP 17.9* 5’-GTGTTCGATCCCTTCTCCCT-3’ and 5’-CGCTGATCTGAAGGACGTTG-3’
*18s* 5’-ACATGCGCCTAAGGAGAAATAG-3’ and 5’-ACCTCCATGCTCACTGGTACTT-3’

### Transcriptomic analysis

Because Zhengdan 958 is a hybrid cross, we chose the cv. B73, which had its genome sequenced, as the material for transcriptomic analysis. Due to the maize cv. B73 dying when treated at 47°C, the high stress temperature was changed to 42°C and the treatment time was increased to 1 day. For transcriptomic analysis, samples of roots were collected from the maize seedlings (cv.B73) previously soaked in guvermectin or water under the described high temperature conditions, as well as the samples from roots that had room temperature treatments. Total RNA was isolated from the samples using Trizol reagent (Invitrogen) and RNA Clean-Up Kit-5 (Zymo Research, R1016), following the manufacturers’ instructions. The extracted mRNA was used to construct the cDNA library, and the library construction was sequenced on an Illumina Hiseq 4000 Platform (Lc-Bio Technologies, Hangzhou, China). The preprocessed transcriptomic reads were mapped to the reference genome of maize cv.B73 using the HISAT package, and the mapped reads of each sample were assembled using the StringTie method. The differentially expressed genes (DEGs) were identified from RNA-Seq data with a cut-off for the corrected p-value < 0.005, using log2foldchange greater than or equal to 1 as a threshold. Analyses of the biological information of DEGs were performed using an online database (http://geneontology.org/).

### Statistical analysis

All experiments were conducted with three biologically independent replicates experiments. Statistical analysis was performed with heteroscedastic test of two-sample t-test (Microsoft Excel), to determine significant differences among samples. P-values of significance represented by * (P < 0.05) and ** (P < 0.01).

## Result

### Effect of guvermectin seed treatment on maize seedling growth and yield

**Figure 1:**
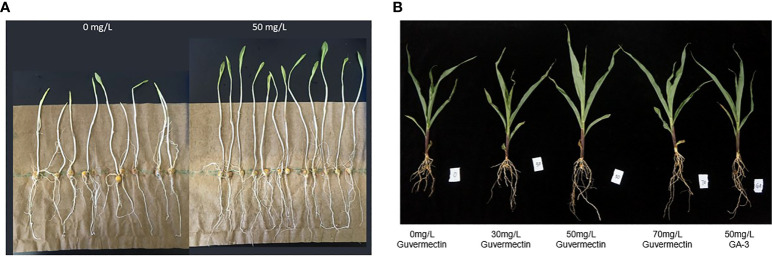
The effect of exogenously applied guvermectin on the maize seedling growth. **(A)** Maize seedling growth after guvermectin treatment in paper roll culture system at 14 days. **(B)** Maize shoot length after guvermectin treatment at 4, 7, 14 days. The experiments were conducted with three biologically independent replicates experiments, per N>24 seedlings per experiment.

Guvermectin treatment was safe in maize seed germination and growth for both the paper roll culture system and in the field (**Figures 1A, B**). After guvermectin treatment, considerable variations in phenotype were observed for seedlings growing in these two culture systems (**Tables 1**, **2**). Firstly, when seeds treated with guvermectin were grown in the paper roll culture system, the growth of the seedlings showed different effects based on the growth period. The shoot height was greater only at 14 days, where shoots from 50 mg/L guvermectin treated seeds were increased by 25.1% compared with the 0 mg/L guvermectin treated seeds (**Figure 1A**; **Table 1**). However, the root length showed no difference (**Figure 1A**; **Table 1**).

**Table 1:** The effects of guvermectin treatment on the maize seedlings growth in paper roll culture system.

Concentration of Guvermectin (mg/L)	Root length (cm)	Shoot length (cm)	Root length (cm)	Shoot length (cm)	Root length (cm)	Shoot length (cm)
	4 d		7 d		14 d	
0	9.50 ± 1.07	8.95 ± 1.63	9.57 ± 1.62	12.21 ± 1.38	11.55 ± 1.41	17.56 ± 2.86
50	9.80 ± 1.28	8.92 ± 1.36	9.85 ± 1.19	13.38 ± 1.72	11.38 ± 1.26	21.88 ± 1.60**

** denotes significance at the 0.01 probability level, respectively. The experiments were conducted with three biologically independent replicates experiments, per N>24 seedlings per experiment.

**Table 2:** The effects of guvermectin treatment on the maize seedlings growth in field condition at seedling stage.

Concentration of Guvermectin (mg/L)	Germination potential (%)	Plant height (cm)	Root length (cm)	Mesocotyl length (cm)	Plant height (cm)	Root length (cm)	Mesocotyl length (cm)	Stem diameter (cm)
			7d			14d		
0	73	21.28 ± 2.42	11.24 ± 1.39	3.04 ± 0.42	39.48 ± 0.95	13.64 ± 2.60	2.00 ± 0.66	9.79 ± 0.61
30 mg/L	76	19.94 ± 1.73	13.00 ± 3.99	2.26 ± 0.56	38.33 ± 3.01	14.11 ± 3.05	1.42 ± 0.54	9.23 ± 1.52
50 mg/L	72	23.93 ± 3.07	13.20 ± 1.82	3.00 ± 0.74	43.05 ± 3.75*	18.35 ± 4.41*	3.20 ± 0.70*	9.77 ± 0.71
70 mg/L	74	21.57 ± 3.49	16.40 ± 1.29*	1.83 ± 0.85	41.23 ± 3.26	16.97 ± 4.07	2.53 ± 1.01	9.52 ± 0.87
50 mg/L GA-3	92	26.14 ± 2.91*	14.76 ± 2.08*	2.74 ± 0.89	43.17 ± 2.56*	15.23 ± 2.59	3.24 ± 0.74*	9.49 ± 0.88

* denotes significance at the 0.05 probability level, respectively. The experiments were conducted with three biologically independent replicates experiments, per N>12 seedlings per experiment.

In field conditions, the growth of maize was affected by guvermectin seed treatment. Guvermectin seed soaking did not promote germination in the same manner as GA-3, and the germination potential was unaffected by different concentrations of guvermectin seed treatment (**Table 2**). However, the plant height, root length, and mesocotyl length of the 50 mg/L guvermectin treated plants were significantly increased at 14 days (P < 0.05) (**Figure 1B**; **Table 2**). Compared with the 0 mg/L guvermectin treated plant, the plant height of seedlings was increased by 9.1%, the root length by 35%, and the length of mesocotyl by 60%. Contrastingly, the stem diameter was unaffected (**Table 2**). Compared with 0 mg/L guvermectin treated plant, the germination, plant height, mesocotyl length of GA-3 treated plants were significantly increased at 14 days.

At maturity, the plant height, stem diameter, and ear height of guvermectin treated plants were not significantly different to the 0 mg/L guvermectin treated plant (**Table 3**). However, the spike weight was significantly increased by 16.7% (P < 0.05) (**Table 3**). The other yield attributes (i.e., the number of spikes and 1000-grains weight) were not significantly different (**Table 3**). Compared with 0 mg/L guvermectin treated plant, the 1000-grains weight of GA-3 treated plants were significantly increased.

**Table 3:** The effects of guvermectin treatment on the maize seedlings growth in field condition at maturity stage.

Concentration of Guvermectin (mg/L)	Plant height (cm)	Stem diameter (cm)	Spike height (cm)	The number of spike per block	Spike weight per block (kg)	1000 grains weight (g)
0	233.75 ± 14.66	24.34 ± 3.95	112.50 ± 11.91	60.67 **±** 6.18	17.34 **±** 1.33	153.00 ± 2.19
30 mg/L	229.80 ± 15.86	24.59 ± 2.41	114.40 ± 9.63	67.33 ± 4.64	19.46 ± 1.51	153.88 ± 1.03
50 mg/L	230.53 ± 24.80	25.55 ± 4.42	113.07 ± 12.13	68.33 ± 4.11	20.24 ± 1.15*	156.75 ± 1.17
70 mg/L	236.20 ± 16.73	23.00 ± 2.28	117.47 ± 10.37	64.00 ± 4.32	17.73 ± 1.92	153.27 ± 3.29
50 mg/L GA	227.27 ± 13.83	24.73 ± 2.18	111.67 ± 11.92	64.00 ± 0.00	18.61 ± 0.56	162.68 ± 2.22*

* denotes significance at the 0.05 probability level, respectively. All samples were recorded at maturity stage.

The experiments were conducted with three biologically independent replicates experiments, per N>60 plants per experiment.

### Effect of guvermectin seed treatment on maize seedlings under varying temperature stress

**Figure 2:**
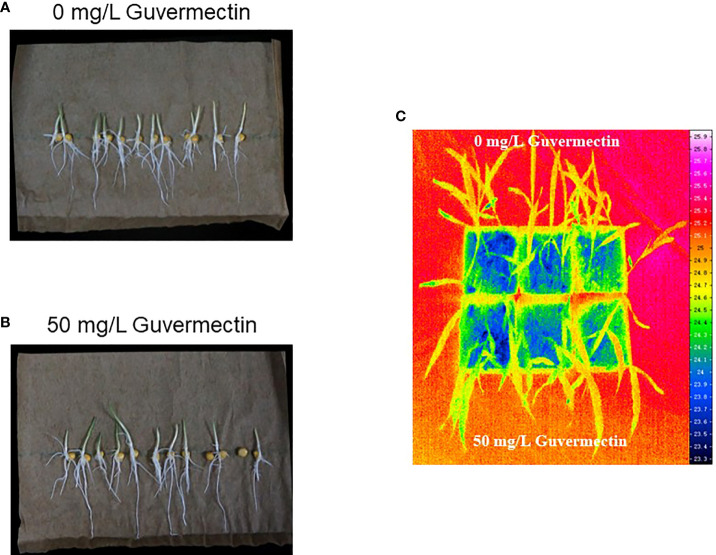
The effect of guvermectin treatment on the tolerance of high temperature at maize seedlings stage. **(A)** Maize seedlings growth after 0 mg/L guvermectin treatment in paper roll culture system under high temperature). **(B)** Maize seedlings growth after 50 mg/L guvermectin treatment in paper roll culture system under high temperature.**(C)** The surface temperature of leaves measured by varioCAM HD infrared camera. The experiments were conducted with three biologically independent replicates experiments, per N>12 seedlings per experiment.

The growth of maize seedlings in paper rolls culture under varying temperature stress were observed. After 50 mg/L guvermectin treatment, the shoot and root length of maize Zhengdan 958 were not significantly different for plants given chilling stress (4°C) or cold stress (10°C), compared to the 0 mg/L guvermectin treatment (**Table 4**). Under high temperature stress (47°C), the shoot length and root length were increased for guvermectin treated seedlings with an increase of 20.65% for shoot length and 23.7% for root length, compared with the 0 mg/L guvermectin treatment (**Figures 2A, B**; **Table 4**).

**Table 4:** The effects of guvermectin treatment on the maize seedlings growth in paper roll culture system under different temperatures.

	0 mg/L Guvermectin		50 mg/L Guvermectin	
	Shoot length (cm)	Root length (cm)	Shoot length (cm)	Root length (cm)
4°C	5.86 ± 0.86	11.00 ± 1.12	4.84 ± 1.27	10.60 ± 1.60
10°C	6.81 ± 1.55	11.76 ± 1.37	6.84 ± 1.49	11.29 ± 2.34
47°C	4.31 ± 0.79	6.90 ± 2.36	5.20 ± 1.54*	8.54 ± 2.32*

* denotes significance at the 0.05 probability level, respectively. All samples were collected from seed germination 7 days.

The experiments were conducted with three biologically independent replicates experiments, per N>12 seedlings per experiment.

At the three-leaves stage of maize in pot culture, the surface temperature was measured by varioCAM HD infrared camera. The surface temperature of leaves treated by guvermectin seed soaking was no different to the control (**Figure 2C**).

### Expression of three small heat shock genes in maize seedlings

**Figure 3:**
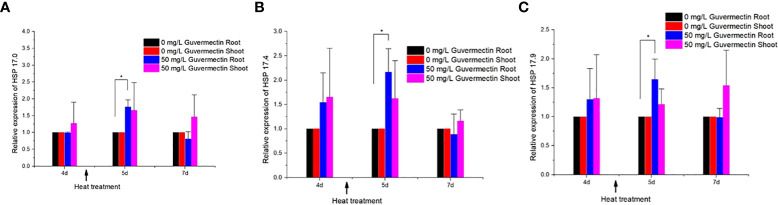
Guvermectin treatment enhanced the expression of HSP 17.0, HSP 17.4, and HSP 17.9 in maize under high temperature. **(A)** The relative expression of HSP 17.0 in maize shoots and roots after guvermectin treatment under high temperature. **(B)** The relative expression of HSP 17.4 in maize shoots and roots after guvermectin treatment under high temperature. **(C)** The relative expression of HSP 17.9 in maize shoots and roots after guvermectin treatment under high temperature. The experiments were conducted with three biologically independent replicates experiments, per N>12 seedlings per experiment. In the graph the values are means ± SE of three independent sets at paper roll cultivation. The arrow indicated the time of heat treatment. * denotes significance at the 0.05 probability level, respectively.

The expression level of these three small heat shock genes in maize shoots and roots, treated with guvermectin, was analyzed under high temperature stress or room temperature. After guvermectin seed treatment, the expression levels of HSP 17, HSP 17.4 and HSP 17.9 were different between shoots and roots under high temperature stress. Guvermectin treatment had no effect on the expression of the three small HSPs in the shoots. In the roots of guvermectin treated plants at room temperature (at 4 days), the expression of HSP 17, HSP 17.4, and HSP 17.9 were not significantly changed compared with the control. Under high temperature, the expression of HSP 17, HSP 17.4 and HSP 17.9 was increased by 1.75, 2.18, and 1.66 times, respectively, in the roots of guvermectin treated plants (**Figures 3A–C**). After transferring the plant to room temperature for 2 days (total 7 days), the expression of HSP 17, HSP 17.4, and HSP 17.9 in guvermectin treated seedling roots was returned to the levels of the control.

### Transcriptomic analysis

Because the HSPs were only more accumulated in roots by guvermectin seed soaking after heat treatment. To further probe the effects of guvermectin at the molecular and cellular level, transcriptomic profiles of roots from guvermectin treated and control plants were compared, under high temperature or room temperature conditions. Among the DEGs in roots under high temperature stress, 133 genes were found to be upregulated in expression, and 30 genes were downregulated after 50 mg/L guvermectin treatment, compared with the 0 mg/L guvermectin treatment. Among the DEGs in roots under room temperature, 362 genes were found to be upregulated in expression, and 599 genes were downregulated after 50 mg/L guvermectin treatment, compared with the 0 mg/L guvermectin treatment. The relative expression of HSP in the transcriptomic analyses were similar when compared with qPCR, showing these analyses was reliable.

Comparing the 163 DEGs under high temperature with the 961 DEGs under room temperature, the number of DEGs in common was only 33 (**Table 5**). The annotation from the GO database showed that the DEGs in common play an important role in the growth of the plant, such as in plant defense, cellulose synthase, carbohydrate metabolism, resistance to stress, terpenoid and aromatics biosynthesis, protein degradation, metal transport, as well as some uncharacterized proteins. These results indicated the effects of guvermectin treatment on the pathways represented by these DEGs genes.

**Table 5:** The common DEGs after guvermectin treatment at high temperature and room temperature.

Transcript id	Description	Fold expression at high temperature	Fold expression at room temperature	Gene function
**Zm00001d031158**	***Pathogenesis-related protein5***	194.51	0.14	**Plant defense**
**Zm00001d048947**	***Pathogenesis-related protein3***	240.89	0.23	
**Zm00001d028816**	***Pathogenesis-related protein6***	15.48	0.19	
**Zm00001d028815**	***Pathogenesis-related protein7***	3.11	0.43	
**Zm00001d028814**	***Pathogenesis-related protein 10***	2.77	0.55	
**Zm00001d034096**	***Cytochrome P450 family 81***	683	0.20	
**Zm00001d049430**	***Germin-like protein subfamily 1, member 8***	3.36	1.95	
**Zm00001d038049**	***Beta-1,3-glucanase 4***	2.46	2.13	
Zm00001d030121	*Roothairless6*	11.81	0.35	Cellulose synthesis
Zm00001d017032	*Glycine-rich cell wall structural protein*	11.73	0	
Zm00001d009309	*Xyloglucan endotransglucosylase/hydrolase 14 (XTH14)*	0.45	0.19	
Zm00001d014987	*Cellulose synthase 1 (hypothetical protein)*	9.51	17.70	
Zm00001d028554	*Cytosolic enolase 3*	2.17	3.89	Glucose metabolism
Zm00001d035038	*Fructinase-2 (FRK2)*	0.33	14.96	
Zm00001d035037	*Fructinase-2 (FRK2)*	2.31	0.36	
**Zm00001d031155**	***Osmotin-like protein OSM34***	280.58	0.16	**Stress response**
**Zm00001d045800**	***C2 and GRAM domain-containing protein***	2.91	2.54	
**Zm00001d016158**	***Prefoldin 6***	0.17	2.51	
**Zm00001d034096**	***Cytochrome P450 family 81***	683.12	0.20	
**Zm00001d044156**	***Cytochrome P450 72A1***	7.15	0.17	
**Zm00001d039310**	***Cytochrome P450 71A26***	4.25	0.29	
**Zm00001d039364**	***DJ-1 homolog B***	4.72	0.14	
**Zm00001d054044**	***Catalase3***	5.74	0.39	
**Zm00001d024210**	***Terpene synthase11***	252.32	0.15	**Terpenoid and benzenoid biosynthesis**
**Zm00001d024207**	***Tps6***	66.78	0.21	
**Zm00001d004624**	***O-methyltransferase ZRP4***	3.48	2.05	
Zm00001d024265	*F-box protein SKIP19*	3.54	2.23	Protein degradation
Zm00001d007753	*Eukaryotic aspartyl protease family protein*	3.26	0.49	
Zm00001d021677	*Aspartic proteinase nepenthesin-1*	4.06	0.45	
Zm00001d015133	*Metal transporter Nramp, NRP6*	2.56	0.48	Metal transporter
Zm00001d046474	*Hypothetical protein*	34.94	0.37	Hypothetical protein
Zm00001d049288	*Uncharacterized protein*	25.95	0.02	
Zm00001d023090	*Hypothetical protein*	0.32	5.74	

## Discussion

Plant growth regulators play an important role in agribusiness. It is necessary that novel, high-efficiency, and inexpensive PGRs are developed for crop production. Guvermectin, as a novel compound, has been registered as a PGR in China. In this study, in the paper rolled culture system, only the shoot length was increased at 14 days after germination, and the root length were not affected. In the field condition, the growth of plant were increased at 14 days by 50 mg/L guvermectin treatment. These results indicated that guvermectin can promote the growth of seedlings. From this study, we found that although guvermectin is an N9-glucoside cytokinin compound, the effects on maize growth after guvermectin treatment were different to that of common PGR treatment. Unlike gibberellin, guvermectin did not affect seed germination (White and Rivin, 2000; Bottini et al., 2004). When applied through seed soaking, cytokinin has previously been found to inhibit roots and promote seedling growth (Kyozuka, 2007; Ramireddy et al., 2021), while auxin inhibit seedling and promote roots growth (Aloni et al., 2006; Overvoorde et al., 2010). Gibberellin can promote seed germination, while ABA inhibits this (White and Rivin, 2000; Zhang et al., 2020). ABA can promote the plant high temperature tolerance by opening and closing of stoma, however, increased high temperature tolerance of guvermectin treated plants was unrelated to their stoma (Mittelheuser and Van Steveninck, 1969; Munemasa et al., 2015). Our results show that guvermectin seed soaking treatment results in terms of germination and seedling growth, and can even improve maize yields in field conditions, indicating its potential use in agribusiness.

We noted a difference in the phenotype of seedlings between field conditions and the paper rolled culture system, suggesting the influence of the different environmental factors, like temperature, water, nutrition and so on. Maize is one of most important crops in China but in many regions the temperature reaches 35–40°C during the seedling growth period, preventing this crop from growing. As the global temperature has risen in recent years (King and Harrington, 2018), one of the visible consequences of a warming world is an increase in the intensity and frequency of extreme weather events (Stott, 2016). Sudden and unexpected high temperature stress is becoming frequent, and can substantially impact plant growth and crop production (Lobell et al., 2011; Bebber et al., 2013; Zhao et al., 2017). In our study, guvermectin treatment shows the potential to alleviate the damaging effect that high temperatures can have on seedling growth. We have demonstrated guvermectin seed soaking treatment can improve the growth and resistance to high temperature stress of maize seedlings, but we did not find any resistance to chilling or cold stress. The guvermectin treated seedlings displayed increased shoot and root length in high temperature environments, which was associated with high temperature field conditions. These results indicated that guvermectin can improve growth by enhancing tolerance of high temperature. Furthermore, by measurement of leaf surface temperature, we found guvermectin was unable to control the stoma opening and closing to improve the tolerance of high temperature as was seen with ABA (Mittelheuser and Van Steveninck, 1969; Munemasa et al., 2015). The changes were similar to the anti-senescent activity of N9-glucoside cytokinin conjugates. This indicated guvermectin has a wide range of agricultural application prospects in the hot Chinese summer.

HSPs are important in the plant response to high temperature stress (Pareek et al., 1995; Shah et al., 2011; Bita and Gerats, 2013). The cDNA of HSP 70, HSP 90, and other HSPs were not be amplified by PCR in our research. Instead, we chose HSP 17, HSP 17.4 and HSP 17.9 as the indicators for the plant response to high temperature stress. We also detected the expression level of small HSP 17, HSP 17.4, and HSP 17.9 in the maize root and shoot under high temperature stress as indicators of high temperature tolerance. We found the expression of the three HSPs in guvermectin treated plants was significantly increased under high temperature conditions only in the roots, with no promotion of HSP expression in the shoots under high temperature stress. Recent studies have reported that high temperature tolerance of plants is increased when overexpressing HSP 17, HSP 17.4, and HSP 17.9 (Sun et al., 2012; Chen et al., 2014; ul Haq et al., 2019). In addition, recent research has demonstrated that small HSPs not only increased the tolerance of high temperature, but also play roles in seed germination (Coca et al., 1994; Chauhan et al., 2012; Kaur et al., 2016). However, guvermectin treatment was unable to increase the expression of the three small HSPs under room temperature conditions, indicating that, while guvermectin regulated plant growth and tolerance, it only indirectly influenced the expression of these HSPs.

Guvermectin seed soaking can promote the growth of maize at seedling stage and increase spike weight in the field and tolerance to high temperature stress. Because the HSP was only more accumulated in roots. We thought the roots was the important place of guvermectin functions we performed the transcriptomic analysis only by roots. In the transcriptomic analyses, we also identified many upregulated and downregulated genes in the roots of guvermectin treated plants, indicating that various pathways were stimulated or inhibited due to this seed soaking treatment. This was performed to determine correlations and patterns that might provide clues for yet unknown functions of guvermectin. It was striking that guvermectin treatment influenced the growth of the maize seedlings. We suggest that this is due to improved tolerance of high temperatures in the maize seedling stage. The transcriptomic data also indicated notable changes in plant defense, cellulose synthesis, carbohydrate metabolism, resistance to stress, terpenoid and aromatics biosynthesis, protein degradation, and metal transport, as well as some uncharacterized proteins. In relation to these genes, cytochrome P450 is implicated in protecting plants from harsh environmental conditions, by mediating biosynthesis of secondary compounds, like plant hormones, terpenes, alkaloids, and other compounds, conferring increased antioxidant activity (Persans et al., 2001; Yan et al., 2016; Castorina et al., 2018; Rao et al., 2020). The pathogenesis related protein family are not only involved in plant immune responses (Wu et al., 2016; Chun and Chandrasekaran, 2019), but also have roles in tolerance of abiotic stresses (Wu et al., 2016; Ali et al., 2018; Desouky et al., 2021). Terpenes and benzenoid compounds were reported as important secondary compounds involved in adaptation to abiotic conditions (Singsaas, 2000; Liu et al., 2021). Together, the changes of these pathways may also influence the tolerance of maize under high temperature stress.

Based on these results, we tentatively explained how guvermectin, an N9-glucoside cytokinin compound considered ineffective in past research, when treating maize seeds, affected the growth of the seedling stage and response to high temperature stress. The upregulation and downregulation of genes by transcriptomic analyses when plants were exposed to high temperature stress suggested a response that relieved damage, and lead to accelerated growth in seedlings under varied environmental conditions. This initial advantage would be influential in reaching the maturity stage and onwards. The advantage at maturity stage was reflected in a corresponding rise in spike weight. As treatment of maize seeds with guvermectin resulted in improved growth at the seedlings stage and tolerance of high temperature stress, guvermectin might be viable as a seed treatment agent in China, or other maize production areas, where temperatures are high during the period of germination and early seedling growth.

## Conclusion

Early conclusions about N9-glucoside cytokinin compounds suggested inactivity in plants. Although guvermectin was a N9-glucoside cytokinin compounds, it can improve the growth of plant, and its activity is different from cytokinin. In this study, guvermectin soaking treatment of maize seeds promoted growth at the seedling stage, increased spike weight, and improved growth of seedlings exposed to high temperature stress. In addition, guvermectin treatment promoted the accumulation of three small heat shock proteins in the plant root. Transcriptomic analysis revealed that genes related to plant defense, stress response, and terpenoid biosynthesis were also regulated in guvermectin treated plants. And the difference of these related pathways possibly improved the growth and tolerance of high temperature stresses of maize. Guvermectin, a new plant growth regulator, has the potential to be an alternative approach to solving the problems of crop growth and production in a warming world.

## Data availability statement

The datasets presented in this study can be found in online repositories. The names of the repository/repositories and accession number(s) can be found below: https://www.ncbi.nlm.nih.gov/, PRJNA872213.

## Author contributions

BZ, XL, WG, WX initiated and designed the research, BZ done the experiments, BZ, CZ, HG, GW, ZH analyzed the data and wrote the manuscript, SZ, MS, YL revised and edited the manuscript and also provided advice on the experiments.

## Funding

We thank the funding provided by National Key Research and Development Programs of China (2017YFD0201602).

## Conflict of interest

The authors declare that the research was conducted in the absence of any commercial or financial relationships that could be construed as a potential conflict of interest.

## Publisher’s note

All claims expressed in this article are solely those of the authors and do not necessarily represent those of their affiliated organizations, or those of the publisher, the editors and the reviewers. Any product that may be evaluated in this article, or claim that may be made by its manufacturer, is not guaranteed or endorsed by the publisher.
